# Small-scale experimental and numerical simulation of blasting in jointed rock-like materials under varied joint and explosive conditions

**DOI:** 10.1371/journal.pone.0333163

**Published:** 2025-10-29

**Authors:** Xuejiao Cui, Mingsheng Zhao, Hongbing Yu, Jianmin Zhou

**Affiliations:** 1 School of Management, Hunan University of Information Technology, Changsha, China; 2 School of Mining, Guizhou University, Guizhou, China; 3 Hongda Blasting Engineering Group Co., Ltd, Guangzhou, China; NIT Raipur: National Institute of Technology Raipur, INDIA

## Abstract

This study focuses on the blasting failure of rock-like materials, aiming to investigate the effect of the joint spatial characteristics and explosive parameters. Rock mass blasting is complex, and the influence of factors like lithology, rock structure, and explosive characteristics needs consideration. In similar model tests, concrete casting molds are used to prepare nine groups of joint models with different joint quantities, angles, and distances. Mixed emulsion explosives are used for blasting, and the crack expansion process and blasting fragmentation properties are analyzed. In numerical simulations, ANSYS/LS-DYNA is employed with carefully selected material parameters. The results show that the blasting effect of rock mass first increases and then decreases with joint width, increases with joint number and spacing, and first increases and then decreases with joint inclination angle. The consistency between the similar model tests and simulation results validates the conclusions of this study, providing theoretical support and practical guidance for optimizing the blasting excavation parameters of the jointed rock mass. This research offers a scientific decision-making basis for dynamic adjustments of blast schemes, safety risk pre-control, and construction efficiency optimization in engineering management.

## 1. Introduction

The rock mass exists within a unique geological environment and is frequently subjected to various influences, including explosions, excavation disturbances, and seismic loads. The damage or failure process of rock under impact loading typically involves the transfer of external energy as well as the dissipation of internal energy. Research indicates that the mechanical properties of brittle materials such as rock exhibit significant differences when subjected to impact loads compared to static conditions; notably, strain rate has a substantial effect on the strength characteristics of rock [1–6]. In recent years, there has been an increasing focus on the dynamic mechanical properties of rocks, concrete, and other materials. Scholars have systematically investigated the energy dissipation mechanisms associated with rock failure under impact loading, resulting in a wealth of research findings [7–11]. Liu et al. [12] proposed an isotropic rock blasting damage model utilizing continuum mechanics and statistical fracture mechanics. The application of X-ray tomography (CT) in the field of rock damage mechanics has enabled the observation and quantification of crack growth within various sections of the rock’s interior. Raynaud et al. [13] employed X-ray CT scanning technology to perform non-destructive imaging of internal cracks in rock samples, investigating their static deformation and fracture processes under three-dimensional stress conditions. Kawakata et al. [14–15] developed three-dimensional CT images based on scanning data from the end faces of rock samples, illustrating both the distribution and morphological characteristics of cracks present within these materials. Huang et al. [16] successfully extracted crack volumes during granite failure under varying strain conditions, thereby providing an effective methodology for microscopically studying the evolution laws governing rock damage. These findings have significantly enhanced the understanding of dynamic properties associated with rocks. However, In the context of real borehole blasting operations, the blasting conditions are inherently complex and variable. Therefore, it is essential to consider the influences of lithology, rock structure, charge configuration, and explosive characteristics. A.K. Raina et al. [17] employed a burst pressure measurement technique to investigate the interaction between explosives and geological formations. The findings indicate that this method effectively elucidates the relationship between explosive properties and rock fragmentation. Additionally, Ajay Kumar Jha and Mesec Josip et al. [18–19] proposed an explosive-rock interaction model grounded in wave impedance theory. Through field tests conducted in a mining environment, they determined the impedances of various rock types and identified the explosives required for optimal rock-breaking efficacy.

Many scholars have adopted numerical simulation methods to study the influence of joints and explosives on the blasting failure characteristics of rock masses [20–25], Including various simulation software and methods, such as PFM [26], XFEM [27], IGA [28] and so on [29–32]. Globally, numerical methods have advanced understanding of jointed rock blasting. M. Stavropoulou et al. [33] first elaborated on a new generalized Poisson probability density function of joint frequencies counted along scanlines and boreholes, which can be used to the Discrete Fracture Network (DFN) modelling of jointed rock masses. Som Nath et al. [34] employs continuum numerical modeling to reveal the coupled control mechanism of Blast-Induced Damage Zones (BIDZ) and joint orientation on rock slope stability along National Highway-5 in Himachal Pradesh. Siamaki Ali et al. [35] employed numerical modeling to quantify blast-induced shear strength degradation in rock discontinuities. He et al. [36] employed PFC software to simulate the effects of blasting stress waves on deeply buried tunnels in the jointed rock mass, and investigated the effects of different values of ground stress and joint angles on tunnel rockburst. Liu et al. [37] used RFPA2D software based on the finite element method and statistical damage theory to simulate the rupture process of rock mass with different joint geometries during the blasting process, and analyzed the influence of factors such as the distance from the joint to the blast hole and the length of the joint on the rupture of the rock mass. Wang et al.[38] used the Holmquist-Johnson-Cook (HJC) intrinsic model in the finite element software LS-DYNA to describe the behavior of rock materials and to study the damage evolution and spatial distribution characteristics of intact and jointed rock bodies under blasting. Liu et al.[39] investigated the effects of different joint development characteristics on the deformation of the tunnel surrounding rock, the destabilization mode, and the force mechanism of rock anchors under the action of blasting vibration utilizing on-site investigation, numerical simulation and on-site monitoring. The results show that the destabilization mode of the tunnel is obviously affected by the larger joint inclination angle.

The dynamic mechanical properties of rock masses exhibit significant variability due to the complex nature of the rock medium and the presence of weak structural planes, such as joints and cracks. This variability is particularly pronounced at different depths and across various rock strata within the same location, which complicates the accurate characterization of the relationship between explosives and rock materials. Consequently, investigating the damage mechanisms in rock masses with diverse structural characteristics under actual blasting conditions represents a critical challenge that must be addressed within the engineering blasting sector.

## 2. Experiment setup

**Fig 1:**
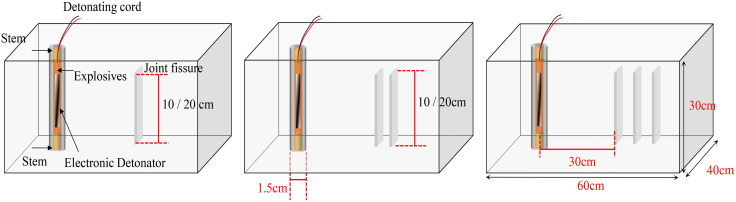
Specimen preparation and design scale.

Concrete was selected as a controlled rock analog due to its homogeneity, reproducibility, and scalability for laboratory testing. While natural rock exhibits inherent heterogeneity, concrete allows systematic isolation of joint geometry and explosive effects. Crucially, concrete’s properties can be calibrated through material composition adjustments to match target rock types, enabling controlled simulation of dynamic fracture behavior. Several sets of concrete casting molds have been developed, and a thorough inspection of the molds is conducted prior to sample pouring to ensure their integrity. Following the inspection, the molds are moistened with water to prevent the wooden boxes from absorbing moisture from the concrete, which could lead to an imbalance in the water-cement ratio. This imbalance may adversely affect both the strength and quality of the concrete, potentially resulting in cracking or spalling. Subsequently, the concrete mixture is poured into the molds. Initially, fine river sand, cement, water, and other materials are meticulously prepared; these components are then added to a mixer according to a specified ratio of ash: sand: water = 1:2:0.5. The dimensions of the sample are illustrated in Fig 1. The measured specimen density was 2162 kg/m^3^. The mechanical results demonstrated a static uniaxial compressive strength of 26.85 MPa. A total of nine distinct groups of joint models are prepared by embedding samples in varying quantities, angles, and distances using hard paper to simulate the closed joint structure of rock masses. The characteristics of the joints include one, two, and three parallel joints measuring 10 cm and 20 cm in length, designated as S-1, S-2, D-1, D-2, T-1, and T-2 respectively. Additionally, a set of joint widths of 10 cm, joint angles of 30°, 60° and 90°, another complete set of samples are used as controls. The sample labels are T-30, T-60, T-90 and Intact.

**Fig 2:**
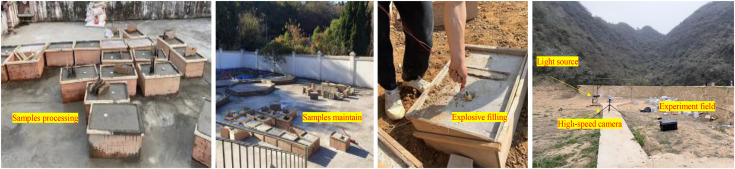
Experimental program and materials.

The prefabricated hole is equipped with a PVC pipe. Stemming utilized dry silica sand compacted to 1.5 g/cm³ density and the thickness of the PVC pipe is 2 mm. After the model is filled with mortar, air bubbles within the cement mortar are eliminated using a vibrator to enhance the compactness of the concrete. As illustrated in Fig 2, once the pouring of the specimen is completed, it will be cured for 28 days under standard conditions. Following this curing period, the demolding of the concrete samples commences. In similar model blasting tests, the mixed emulsion explosives were sensitized on-site. All samples used explosives from the same batch, which were applied within 30 minutes of sensitization, and pre-test verification showed that their velocity of detonation (VOD) was 4100 ± 50 m/s. Physical sensitization ensured temporal stability, thereby avoiding the effects of chemical degradation. To guarantee consistent explosive performance across different batches, the preparation of mixed emulsion explosives adheres to uniform emulsification and sensitization parameters. Additionally, detonation velocity tests (using an instantaneous detonator) indicate that the detonation speed of the mixed emulsion explosive is approximately 4110 m/s. Given the substantial volume of each sample and to achieve optimal blasting effects, each hole’s charge is limited to 20 g, with an electrical detonator placed in per hole.

## 3. Experiment results analysis

### 3.1. Cracks expansion process

**Fig 3:**
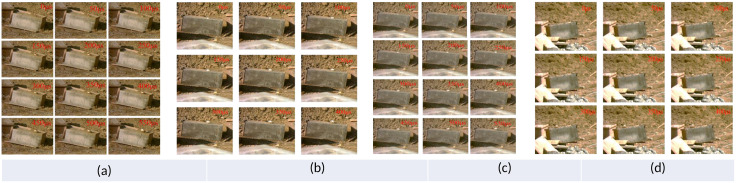
Fracture process of different number of joints under blasting. **(a) S-1; (b) S-2 (c) D-1;** (d) **D-2.**

Crack propagation was recorded at 36,000 fps using a FASTCAM SA1.1 high-speed camera with 512 × 512 resolution. The system was triggered by the detonator current and the frame rate of the high-speed camera is set at 36,000 fps, which corresponds to a maximum acquisition time of 2.77 s, and the crack expansion process of samples can be clearly observed at this frame rate. Fig 3 presents a detailed overview of the damage and failure mechanisms observed in specimens with single and double joints subjected to explosive loading. In Fig 3(a), the propagation process of explosive-induced cracks is illustrated under the condition of a single joint measuring 1 cm. At 50 μs, loosening occurs at the blast hole, leading to an ejection of material from the PVC pipe. By 100 μs, a vertical crack emerges and continues to propagate thereafter. It is not until 400 μs that new cracks begin to manifest on the side of the specimen. At this stage, the sample primarily experiences effects from explosive gases, resulting in microcracks that propagate and widen. Fig 3(b) illustrates the propagation of a 2 cm wide crack within a single joint. Following the explosion of the explosive, deformation occurs in the blast hole, resulting in the ejection of the PVC tube from the hole due to impact from the explosion. At 150 μs, a vertical crack emerges on one side of the sample and penetrates through its entirety, subsequently leading to branching cracks. After 250 μs, no further increase in crack quantity is observed; however, their width continues to expand. The overall number of generated cracks remains relatively low, indicating that explosive gas takes longer to escape and exerts prolonged pressure on the borehole wall, thereby ensuring optimal utilization of explosive energy. Figs 3(c) and 3(d) show the explosive crack propagation process of specimens with a double section width of 1 cm and 2 cm, respectively. For samples with shorter widths, no obvious cracks are observed within 200 μs after explosion, only PVC pipes are observed to spray out after being impacted. At 250 μs, cracks begin to appear, but the number is small and gradually penetrates the side of the sample without branching cracks. Around 500 μs, smoke and mud begin to emerge from the borehole, and a large amount of explosive gas is released. Afterward, fragments of the sample gradually form. The failure process of the 2 cm width double section results in slight deformation of the sample surface after explosion, and no other phenomena are observed. Until 150 μs, cracks began to appear on the side of the sample, followed by branching cracks in the middle of the sample. At 300 μs, the cannon smoke begins to emerge, the crack width increases, and the expansion is basically completed.

**Fig 4:**
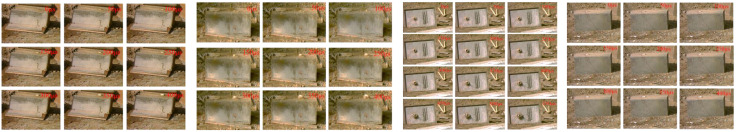
Fracture process of different angles of joint under blasting. (a) T-30; (b) T-60 (c) T-90; (d) Intact.

Fig 4 shows the damage and failure snapshots of three joint specimens with different inclination angles and a control group specimen without joints under blasting load. For the specimen with a three-joint inclination angle of 30 °, as shown in Fig 4(a), fine cracks begin to appear at 50 μs and propagate rapidly. At around 150 μs, a branching crack appears in the middle of the sample side. The cracks in the lower part are fully developed, intersecting, and the number of blocks is increasing. Around 400 μs, no new cracks are generated and the crack width continues to increase. During this process, the slow emission of explosive gas indicates that the explosive has done sufficient work for a long time, and more energy is used to crush the concrete sample, improving the energy utilization efficiency of the explosive. However, for the sample of 60° inclination shown in Fig 4(b), the PVC pipe in the hole after explosion deformed after being impacted, and the surface crack of the sample is not formed at this time. After 150μs, a crack appeared at the top of the specimen side. At 200μs, the cracks spread through the whole specimen and branch cracks appeared in the middle of the specimen. At 300μs, a new crack appeared on the left side of the crack, and then gradually produced some fine cracks. After 400μs, the crack width increases, and the number of cracks basically does not change. In Fig 4(c), crack propagation on the specimen surface can be clearly seen. After explosion, a slight deformation occurs on the specimen surface within 100μs, most notably around the hole. At 150μs, two cracks appear around the hole and continue to expand towards the free plane. Since then, the number of cracks continues to increase, and the whole is radial. At 250μs, the crack spread to the joint. However, it does not expand directly through the joint, but continues to expand at the edge of the joint. This shows that the joint crack hinders the crack propagation and changes the direction of stress wave propagation. Since then, the number of cracks does not increase, the width of cracks continues to increase, and the smoke and blockages emerge from the gun holes. Finally, when the crack growth process of the control group is 150μs, the PVC pipe has an obvious displacement, and the upper surface of the sample also has an obvious deformation. At 250μs, the obvious crack growth is seen, and the expansion speed is faster. At 350μs, the crack generation has basically ended, the number of cracks is large, and the crack width keeps increasing. During the whole explosion process, the smoke from the gun is emitted for a long time. At about 600μs, new cracks no longer occur, and smoke and blockages begin to emerge as the width increases. This shows that the action time of the explosive gas is longer, the explosive energy is fully utilized, and it is conducive to the crushing of the sample.

In summary, when an explosion occurs in the rock mass, it generates a strong shock wave. Under the action of this shock wave, the surrounding rock mass is subjected to impact and compression, resulting in the local crushing of the rock mass. After passing through the ground area, the pressure wave diffuses further, but its strength is already weakened and cannot directly cause the rock to break. Once the pressure wave reaches the free surface, it is reflected and forms a tensile stress wave. Since the tensile strength of the rock is much less than its compressive strength, if the strength of the reflected stress wave exceeds the dynamic tensile strength of the rock, the rock will start from the free plane and pull apart layer by layer along the direction of the explosion source. As the reflected wave travels in the direction of the blast source, the phenomenon of falling will continue to occur until all rocks within the range of the explosion have been destroyed. The free-surface crack is mainly generated in the area closest to the hole, because the stress wave arrives there first and produces the emission stretching effect, and the stress concentration occurs, resulting in the crack. Subsequently, under the action of explosive gas, the crack continues to expand, and the width continues to increase. At this time, explosive gas and blockage can be clearly seen pouring out of the gunhole.

### 3.2. Blasting fragmentation properties

**Fig 5:**
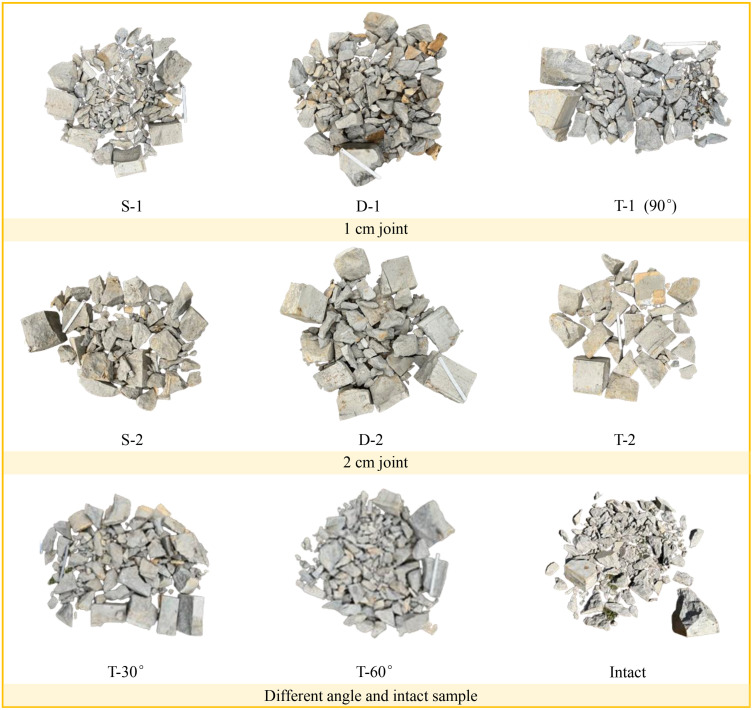
Fragment images for different numbers and angles of the joints in rock mass.

There are cracks in materials such as rock masses, which affect the propagation of stress waves. Conduct corresponding fragmentation analysis for blasting tests with different joint widths and angles, with the main parameters studied being X_50_ and X_100_, where X_50_ reflects the average size of the fragments; X_100_ is the maximum size of the fragments, which can well describe the large block rate generated by the explosion. The fragment morphology of the sample after blasting is shown in Fig 5.

A large amount of measured data and literature indicate that the five-parameter Swebrec modified distribution function can well describe the distribution law of rock blasting fragments. Therefore, this article, after obtaining the sizes of each rock block through ImagJ image processing, uses the Swebrec modified distribution function to obtain the size distribution curves for each group of rock blasting tests. The basic expression of this function is as follows:


$$P(x)=1/\left\{1+A\left[\ln(x\max x)/\ln(x\max x_{50})\right]B+(1-a){\left[\left(\frac{x_{\max}}{x}-1\right)/\left(\frac{x_{\max}}{x_{50}}-1\right)\right]}^{C}\right\}$$ (1)

where *x*_max_ is the maximum size at P(*x*_max_) = 100%, which can be obtained by directly measuring the size of the largest fragment; *x*_50_ is the median size at P(*x*_50_) = 50%; A is the grading coefficient, with a range of values from 0 to 1; and B and C are the fitting parameters, with a range of values around 2.

**Fig 6:**
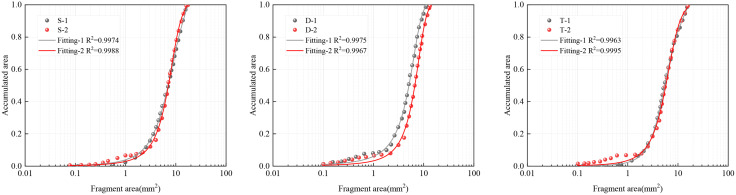
Fitted curves of the distribution pattern of the blasting block size for each group of specimens: (a) Single joint; (b) Double joints; (c) Three joints.

The block size distribution of samples with different joint widths and numbers after blasting is shown in Fig 6 (a) – 6(c), and the fitting effect is good, with a variance R^2^ greater than 0.99. The three groups of tests show a consistent rule, that is, when the joint block degree is 1 cm, the blasting effect is better, and the average size of the fragments is smaller. From the point of view of *x*_50_ of each group of experiments, there is not much difference, and they are all within 1 cm, which is more consistent with the theory. The joint angle will affect the fracture mode of rock and the formation of mass in the blasting operation. If the Angle of the joint is inconsistent with the direction of the blasting, the blasting shock wave may propagate along the joint plane, causing the block to split unevenly or break in an unexpected direction. When the joint dip Angle is large, the shock wave generated by the blasting operation may be more easily concentrated at the junction of the joint surface, thus increasing the probability of rupture in these areas. This can lead to the formation of larger clumps near the junction of the joint surfaces. Joint inclination also affects the area of rock fracture. When the joint inclination is large, it may be relatively difficult for the fracture to expand in the rock, which may result in a smaller fracture area and smaller block formation.

**Fig 7:**
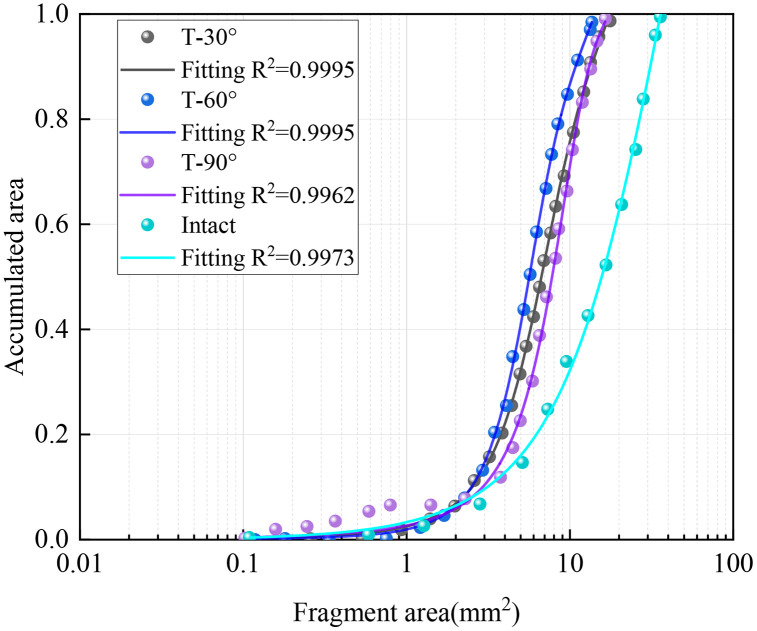
Fitted curves of the distribution pattern of the blasting block size for comparison specimens.

Fig 7 shows the distribution pattern of blasting fragmentation for three joints with different inclinations and intact rock masses. From the analysis of the distribution of blasting fragmentation, when the joint inclination angle is 90 °, the average fragment size is the largest, and the average fragment size is the smallest at 60 °. When the joint inclination angle is large, the propagation of stress waves is hindered, and some of them become reflected tension waves, which weakens the effect of stress waves on the medium, so the blasting effect is worse. When the joint angle is 30° or 60°, the stress wave is reflected to other free surfaces, further enhancing the damage effect. When there are more joints, there are more fracture paths in the rock. The large number of joints may cause the energy to disperse between the different cracks. This can make it impossible to concentrate energy on one rupture path, resulting in relatively small clumps. A higher number of joints means that there are more cracks, which can lead to an increase in the fracture area. More fissures provide more fracture surfaces, allowing the rock to break in more areas, resulting in more burst blocks. In addition, according to the lumpiness distribution curve, with the increase of the number of joints, the curve gradually rises, the X_50_ constantly decreases, and the average fragment size gradually decreases. The different number of joints form complex fracture patterns in the rock, which affects the formation of burst blocks, making the distribution and size of the blocks more diverse.

## 4. Numerical models and simulation analysis

### 4.1. Modeling and parameter selection

Based on the experimental results, the finite element software ANSYS/LS-DYNA is further employed to investigate the influence of joints and explosives on the failure characteristics of rocks. The Lagrangian method with duplicate nodes is used to set up the explosive and rock materials. Meanwhile, an additional void material needs to be defined to provide spatial for the volume expansion of the explosive. The void material is defined using exactly the same material parameters as those of the explosive material. To obtain the material parameters, the mechanical properties are tested in the model tests by taking samples in the field and making standard specimens. The TAW-2000 triaxial testing apparatus is employed to conduct a series of tests, including uniaxial compression tests, Brazilian splitting tests and direct shear tests. Subsequently, the measured rock mechanical parameters are entered as the requisite rock material parameters for the ensuing numerical simulation. The rock material is predominantly defined by means of the material keyword MAT_PLASTIC_KINEMATIC, which represents the plastic kinematic model material. The constitutive model of this material is capable of mirroring the elastic and plastic failure processes that occur in media like soil, rock, and concrete when subjected to explosive blasting. In light of the mechanical test outcomes of cement mortar specimens from similar model tests, the specific parameters including density *ρ* = 2000 kg ∙ m^3^, elastic modulus *E* = 2.60 GPa, compressive strength *σ* = 4.65 MPa, tensile strength *σ*_*t*_ = 8.4 MPa, Poisson’s ratio **v* *= 0.23. MAT_ADD_EROSION enables the definition of a variety of parameters associated with material failure, encompassing compressive stress, tensile stress, equivalent stress, strain, and failure time. In the context of the MAT_PLASTIC_KINEMATIC material, it suffices to utilize this keyword merely for specifying the tensile strength [40]. Throughout this simulation, for every model in which the tensile strength parameter of MAT_ADD_EROSION has been defined, said parameter is uniformly set to −0.47 MPa (notably, the tensile stress in MAT_ADD_EROSION is denoted with a negative sign). MAT_HIGH_EXPLOSIVE_BURN material is utilized for defining both high-energy explosives and void materials. In the present study, mixed emulsion explosives that match the explosion performance of similar model tests are employed. These explosives possess a density of 1050 kg/m³ and a detonation velocity of 4200 m/s. In addition to the basic material parameters, when specifying explosives within the ANSYS/LS-DYNA software environment, it is imperative to also conFig the parameters of the *EOS_JWL equation of state [41]. The EOS_JWL equation of state is as follows:


$$P=A[1-\frac{\omega }{R_{\text{1}}V}]e^{-R_{\text{1}}V}+B[1-\frac{\omega }{R_{\text{2}}V}]e^{-R_{\text{2}}V}+\frac{\omega E}{V}$$ (2)

where *A*, *B*, *R*_1_, *R*_2_, and *ω* are the parameters related to the explosive determined by experiments [41]; *V*_0_ is the relative specific volume, *E*_0_ is the initial specific internal energy. The specific parameters of the explosive’s equation are shown as Table 1.

**Table 1:** Specific parameters for JWL equation.

*A*	*B*	*R*_1_	*R*_2_	*OMEG*	*E*_0_	*V*_0_
2.144E + 11	0.182E + 9	4.2	0.9	0.15	4.192E + 9	1.0

Corrugated paper is utilized as filling material to mimic joint fissures. In static mechanical simulation scenarios such as those involving corrugated paper in carton drop tests, it is a common practice to assume the material as anisotropic elastic. In this study, considering that the dynamic mechanical effect induced by explosives differs markedly from the hydrostatic scenario and that the corrugated paper is employed to mimic joints, it can be further streamlined and modeled as a linear elastic material. The specific parameters density *ρ* = 600 kg ∙ m^3^, elastic modulus *E* = 30MPa, Poisson’s ratio **v* *= 0.3. The size of numerical simulation models aligns with those of similar models and joints are segmented and formed utilizing cutting bodies.

**Fig 8:**
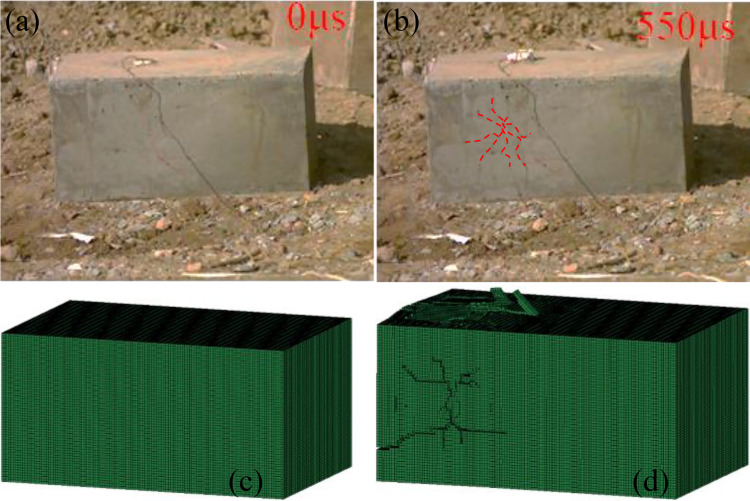
Comparison of morphologies between intact sample blasting test results and simulation results.

Fig 8 presents a comparison of the morphologies between the blasting test results and simulation results for intact samples. In the experimental blasting, at 0 μs, no initial crack exists in the specimen. By 550 μs, the crack propagates and exhibits a radial pattern. In the numerical simulation, the initial model is devoid of cracks; however, post – blasting, a crack propagation path emerges internally. The direction and general distribution of crack propagation in both the experimental and simulation models show similar trends, reflecting the characteristic of cracks extending outward from the detonation zone under the action of blasting energy. After the experimental blasting, distinct damage and crack distribution regions are observable on the specimen surface. The numerical simulation model also reveals internal damage and failure regions, which correspond to the experimental results in terms of damage extent and concentration. Thus, the validity of the numerical simulation parameters is verified.

### 4.2. Effect of joints on blasting fragmentation and equivalent stress distribution

**Fig 9:**
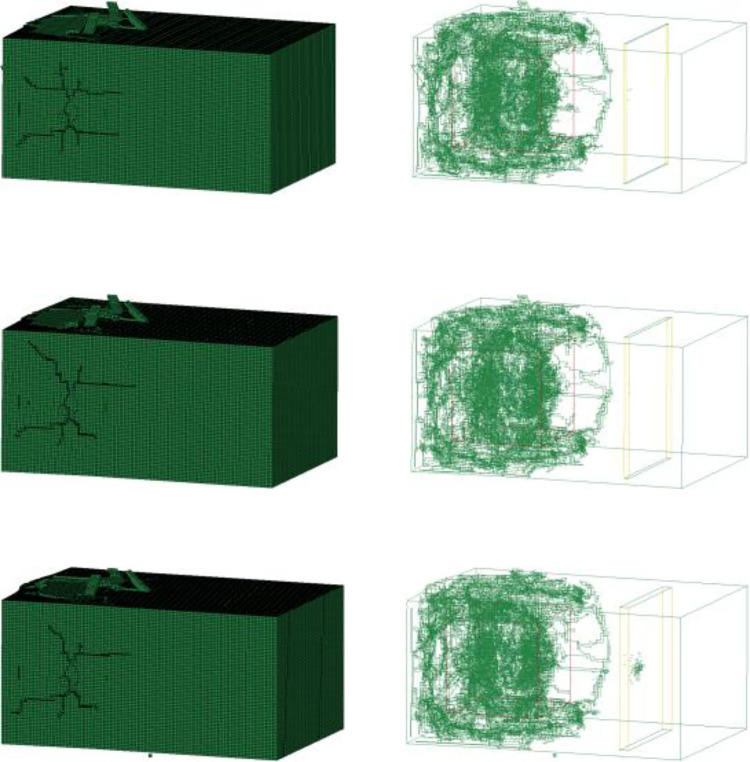
Distributions of failed elements in the blasting models with a single joint: (a) Joint width of 5 mm; (c) Joint width of 10 mm; (c) Joint width of 20 mm.

In similar model tests, the average fragmentation size X_50_ and the maximum fragmentation size X_100_ data are used to conduct a comparative analysis of the blasting effects under different conditions. In this study, the ratio of failed elements is defined as the ratio of the number of failed elements to the total number of elements. It is used to reflect the impact of different condition changes on the rock-mass failure effect. A large average fragmentation size of a rock mass implies that the rock is not broken up thoroughly enough and that fewer parts of the rock mass are destroyed. The average block size has an opposite correspondence with the fail element. Therefore, the consistency of the laws between the two can be analyzed by comparing the average fragmentation size data obtained from the tests with the number of failed elements in the numerical simulation. Fig 9 shows the distributions of failed elements in the blasting models with joint widths of 5, 10, and 20 mm.

**Fig 10:**
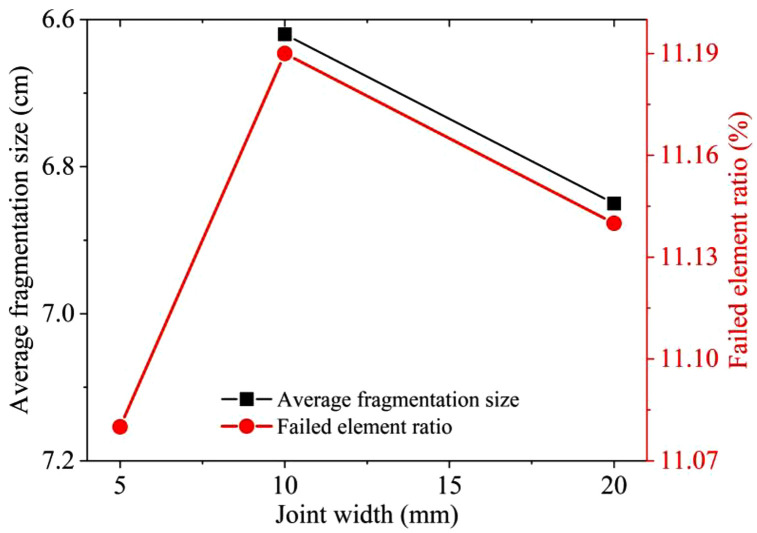
Comparison between similar tests and numerical simulations of different joint widths.

The results of the similar model tests and the numerical simulation are shown in Fig 10. In similar model tests, the average fragmentation sizes X_50_ for single joint widths of 10 mm and 20 mm are 6.62 cm and 6.85 cm respectively. In the numerical simulation, the failed element ratio for single joint widths of 5 mm, 10 mm, and 20 mm are 11.08%, 11.19%, and 11.14% respectively. It can be observed that the results of similar tests and numerical simulations exhibit consistent patterns. Under the three joint width conditions of 5 mm, 10 mm, and 20 mm, the number of failed blasted rock masses is the largest under the 10 mm condition, accounting for the largest ratio of the model rock mass elements. Similarly, under similar model test conditions, the average fragmentation size is the smallest when the joint width is 10 mm. The existence of joint fissures in the rock mass provides an area for the blasting stress wave to enhance stress concentration in the damaged area, and the destructive effect of the stress wave around the joint area is also affected by the joint width. Judging from the results of similar model tests and the numerical simulations, the influence of joint width on the blasting effect is relatively small, and it first increases and then decreases with the change of width. When the joint width is within a certain range, the blasting fragmentation and damage effect of the rock mass is better compared to other widths. Among the several conditions of the similar test and numerical simulation results in this study, the blasting effect is the best when the joint width is 10 mm.

**Fig 11:**
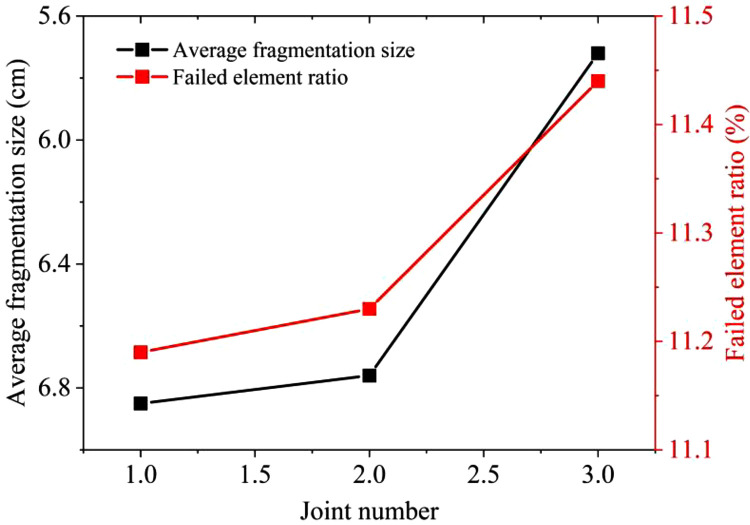
Comparison between similar tests and numerical simulations of different joint numbers.

The results of both the similar model tests and numerical simulations are presented in Fig 11. In the similarity model tests, the average fragmentation sizes X_50_ of one to three sets of 1 cm thick joints are determined to be 6.85 cm, 6.76 cm and 5.72 cm respectively. For numerical simulations, the failed element ratio corresponding to one to three sets of 1 cm thick joints are 11.19%, 11.23%, and 11.44% respectively. The results of similar model tests and numerical simulations exhibit consistent patterns. As the number of joints increases, the blasting-induced damage to the rock mass gradually becomes more significant. Under the condition of a single joint, the number of failed rock mass elements after blasting is close to that of the intact model. The presence of a single joint mainly affects blasting by potentially enhancing the blast-reflected tensile damage effect, and the effect is related to the distance between the blast source and the joint. With an increase in the number of joints, under the conditions of two and three joints, the existence of joints reduces the strength of the rock mass. The reflection of stress waves between joints also contributes to the damage effect in the fissure area. Therefore, as the number of fissures increases, more fracture surfaces are formed in the rock mass, which is conducive to the damage effect of the rock mass. The variation in the number of failed rock mass elements in the simulation is consistent with the variation law of the fragmentation size after blasting, as statistically obtained from similar model tests.

**Fig 12:**
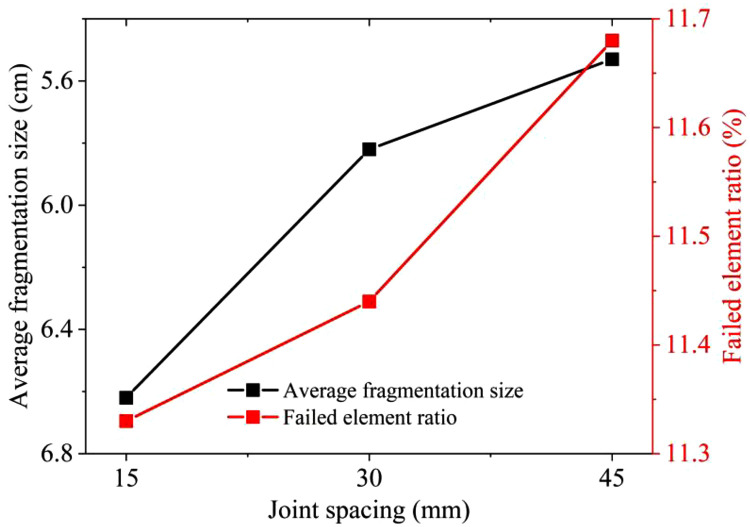
Comparison of similar tests and numerical simulations with different joint spacings.

Fig 12 shows the comparison of similar tests and numerical simulations with different joint spacings. In the similarity model tests, the average fragmentation sizes X_50_ for joint spacings of 15 mm, 30 mm, and 45 mm are 6.62 cm, 5.82 cm, and 5.53 cm respectively. In numerical simulations, the failed element ratio for 1 cm thick joints with spacings of 15 mm, 30 mm, and 45 mm are 11.33%, 11.44%, and 11.68% respectively. The results of the similar model tests are close to those of the numerical simulations. Under the three conditions of different joint spacings of 1.5 cm, 3.0 cm, and 4.5 cm, as the joint spacing increases, the number of failed rock mass elements continuously increases. Compared with the influence of the number and width of joints, the influence of joint spacing is relatively more significant.

**Fig 13:**
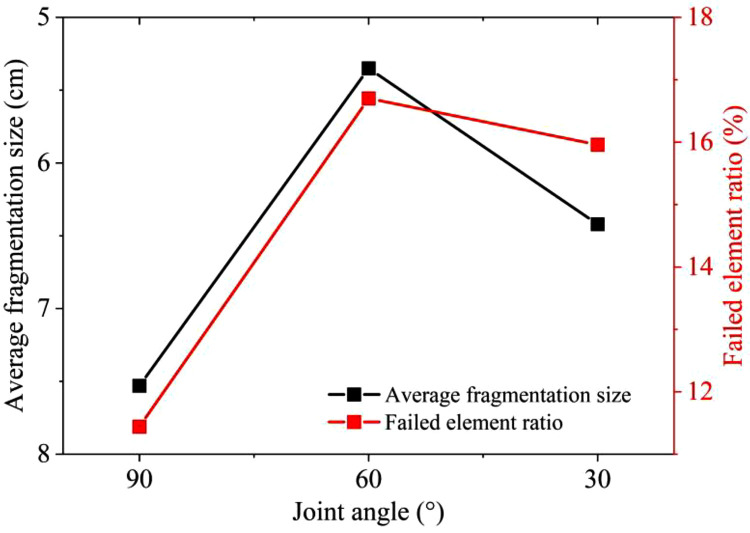
Comparison of similar tests and numerical simulations with different joint inclination angles.

The comparison of similar model tests and numerical simulations with different joint inclinations is shown in Fig 13. In similar model tests, the average fragmentation sizes X_50_ for joint inclinations of 90°, 60°, and 30° are 7.53, 5.35, and 6.42 cm respectively. In numerical simulations, the failed element ratio for 1 cm thick joints with the inclination angles of 90°, 60°, and 30° are 11.44%, 16.70%, and 15.96% respectively. The results of the similarity model tests are consistent with those of the numerical simulations. Under the three joint inclination angle conditions, as the angle between the joint and the central axis of the rock model decreases, the number of failed rock elements first increases and then decreases, reaching its maximum at an inclination angle of 60°. In terms of the simulation results, when the joint inclination angle is 90°, the blasting stress wave is closer to perpendicular incidence on the joint surface. The majority of the stress wave energy is reflected in the blast source. Since the fragmentation effect is most intense in the area near the blast source, this reflection enhances the blasting effect in the blast-source area but has a limited impact on increasing the overall blasting effect of the rock mass. Under the inclination angle conditions of 60° and 30°, more of the blasting stress wave energy is obliquely incident and reflected towards the free surface area of the model. The reflection of the stress wave between the joint surface and the free surface can further strengthen the fragmentation effect of the rock mass near the joint, thus improving the overall blasting and fragmentation of the rock mass. When the joint inclination angle is 30°, due to the relatively large angle, the reflection surface for the blasting stress wave is smaller compared to that at 60°. As a result, compared to the inclination angle of 60°, the energy of the stress wave reflected between the joint and the free surface is reduced.

### 4.3. Effect of mixed emulsion explosives on blasting fragmentation of jointed rock

**Fig 14:**
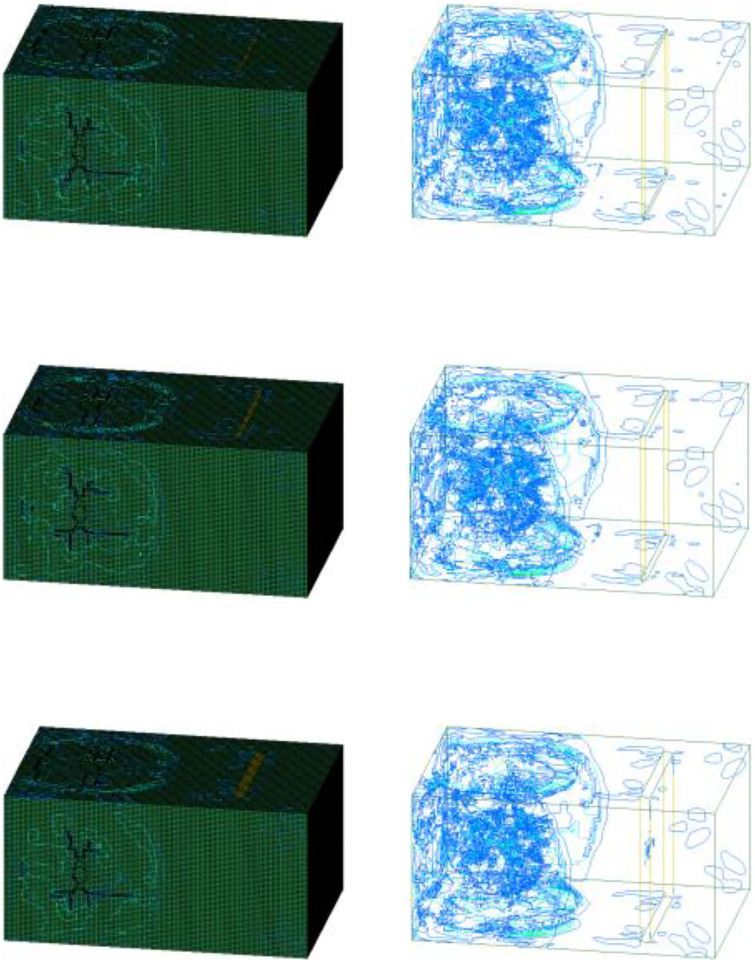
Von Mises stress of the blasting model with a single joint under H1 mixed emulsion explosive: (a) Joint width of 5 mm; (c) Joint width of 10 mm; (c) Joint width of 20 mm.

Three types (H1, H2, H3) of mixed emulsion explosives, which possess different detonation velocities, densities, and brisance, are selected to conduct a statistical analysis of the blasting effects. By analyzing the blasting effects under different conditions, the failure of rock elements in the numerical model and the equivalent stress during the blasting process is obtained. Thus, the development and final failure effect of rock mass damage during the rock-blasting simulation can be determined. The dimensions and the blasting parameters of the numerical models remained consistent, differing only in the explosive properties. The detonation velocities and brisance of H1, H2, H3 mixed emulsion explosives are 4200 m/s, 3800 m/s, 3600 m/s and 16.1 mm, 15.7 mm, 15.3 mm respectively. Three types of mixed emulsion explosives have the same density of 1.12 kg/m^3^. The simulation results of different types of mixed emulsion explosives under different joint widths are obtained by varying the explosive parameters. Fig 14 illustrates the distributions of Von Mises stress in the blasting models where the joint widths are 5 mm, 10 mm, and 20 mm when using the H1 mixed emulsion explosive.

**Fig 15:**
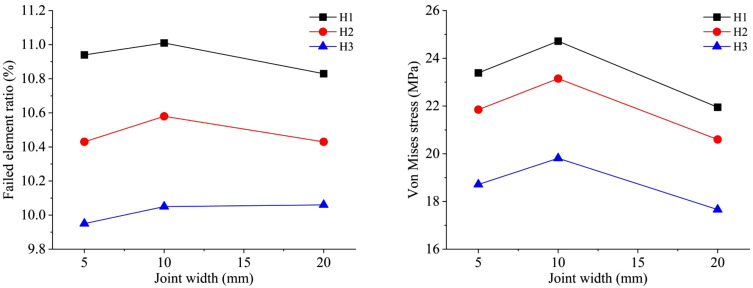
Numerical results of blasting model with a single joint under three types of mixed emulsion explosive: (a) failed element ratio; (b) Von Mises stress.

Fig 15 shows the numerical results of the failed element ratio and Von Mises stress under different types of mixed emulsion explosives. It can be observed that the numerical simulation results of the three types of mixed emulsion explosives exhibit a consistent pattern. The variation in the joint width of the rock mass has a relatively small impact on the blasting effect, and the overall trend shows an initial increase followed by a decrease. The existence of joints and fractures in the rock mass provides a stress concentration area for the blasting stress wave, and the damaging effect of the stress wave around the joint area is also affected by the joint width. Under the conditions of joint widths of 5 mm, 10 mm, and 20 mm, when using the H1 mixed emulsion explosive, the number of failed elements is the largest, accounting for the highest ratio of the rock elements in the model. When the joint width is 10 mm, the ratios of failed elements corresponding to the H1, H2, and H3 mixed emulsion explosives reach their maximum values of 11.01%, 10.58%, and 10.05% respectively. Under different joint width conditions, the overall variation trend of the Von Mises stress is firstly increasing and then decreasing. When the type of explosive changes while other conditions remain the same, the overall trend of Von Mises stress is: H1 > H2 > H3. Moreover, the average Von Mises stress values reach their maximum at a joint width of 10 mm and the blasting effect is the best. At this time, the Von Mises stress values corresponding to the three types of mixed emulsion explosives are 24.72 MPa, 23.15 MPa, and 19.81 MPa respectively.

**Fig 16:**
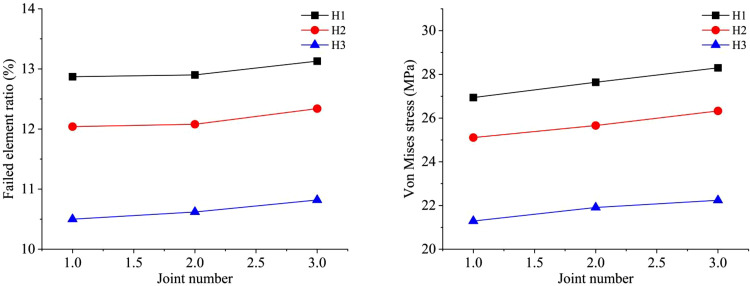
Numerical results of blasting model with different joint numbers under three types of mixed emulsion explosive: (a) failed element ratio; (b) Von Mises stress.

By altering the explosive parameters, the simulation effects of different types of mixed emulsion explosives under different numbers of joints are obtained. The numbers of joints are set as 1, 2, and 3 respectively. The simulation results of the failed elements ratio and Von Mises stress in the models are presented in Fig 16. Under different numbers of joints, the failed element ratio increases with joint number. When the type of explosive changes while other conditions remain constant, the overall trend of the failed element ratio remains as: H1 > H2 > H3. When the joint number is 3, the failed element ratio in all working conditions reaches the maximum value and the blasting effect is the best. At this time, the values of the failed element ratio corresponding to the three types of mixed emulsion explosives are 13.13%, 12.34%, and 10.82% respectively. The variation trend of the Von Mises stress of elements is identical to that of the failed element ratio. Generally, both show an increasing trend as the joint number rises. When the type of mixed emulsion explosive changes while other conditions remain unchanged, the overall trend of the Von Mises stress persists as: H1 > H2 > H3. The average Von Mises stress value reaches its maximum when the joint number is 3, indicating the optimal blasting effect. Von Mises stress values corresponding to the three types of mixed emulsion explosives are 28.30 MPa, 26.33 MPa, and 22.24 MPa respectively.

**Fig 17:**
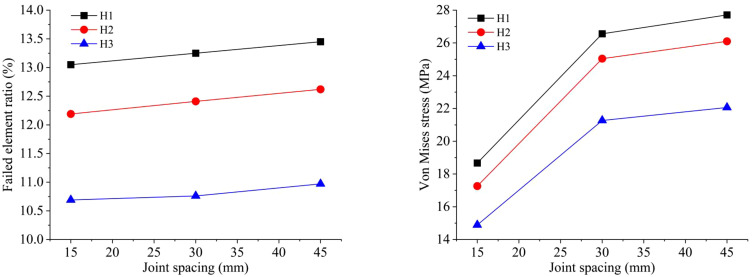
Numerical results of blasting model with different joint spacings under three types of mixed emulsion explosive: (a) failed element ratio; (b) Von Mises stress.

By changing the explosive parameters, the simulation effects of different types of mixed emulsion explosives under different joint spacings are obtained. The joint spacings are set at 15 mm, 30 mm, and 45 mm respectively. As shown in Fig 17, under different joint spacing conditions, the failed element ratio in the numerical simulation model increases with the joint spacing. When the type of explosive changes while other conditions remain the same, the overall trend of the failed element ratio remains as: H1 > H2 > H3. When the joint spacing is 45 mm, the failed element ratio reaches its maximum value and the values of the failed element ratio corresponding to the three types of mixed emulsion explosives are 13.45%, 12.62%, and 10.97% respectively. Under different joint spacing conditions, the variation of Von Mises stress of elements is similar to that of the failed elements ratio. Overall, both show an upward trend as the joint spacing increases. When the type of mixed emulsion explosive is altered while other conditions remain constant, the overall Von Mises stress trend remains as H1 > H2 > H3. The average Von Mises stress reaches the peak value when the joint spacing is 45 mm, indicating the optimal blasting effect. At this moment, the Von Mises stress values corresponding to the three types of mixed emulsion explosives are 27.71MPa, 26.10MPa, and 22.06MPa respectively.

**Fig 18:**
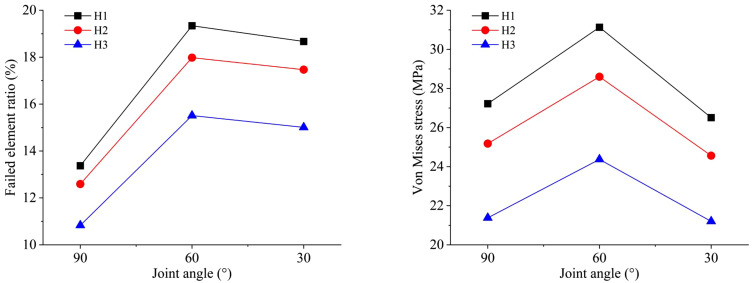
Numerical results of blasting model with different joint inclination angles under three types of mixed emulsion explosive: (a) failed element ratio; (b) Von Mises stress.

As shown in Fig 18, the joint inclination angles are set at 30°, 60°, and 90° respectively. When the joint inclination angle is 60°, the failed element ratio is the largest, and the degree of damage is the most severe. Next is the case of 30°, and then the case of 90°. The failed element ratio in the three conditions is higher than that in the intact model. When the type of explosive changes while other conditions remain the same, the overall trend of failed element ratio remains as: H1 > H2 > H3. When the joint inclination angle is 60°, the failed element ratio reaches its maximum value and the blasting effect is the best. The values of the failed element ratio corresponding to the three types of mixed emulsion explosives are 19.34%, 17.98%, and 15.51% respectively. Under different joint inclination angle conditions, the variation of Von Mises stress in the elements is similar to that of the failed element ratio. When the joint inclination angle is 60°, the Von Mises stress is the largest, indicating the most severe damage. Next is the case with a joint inclination angle of 30°, followed by the joint inclination angle of 90°. The Von Mises stress inthree joint inclination angle cases is larger than that in the intact model. When the type of mixed emulsion explosive is changed while other conditions remain constant, the overall trend of Von Mises stress remains as H1 > H2 > H3. The value of average Von Mises stress reaches its maximum when the joint inclination angle is 60°, meaning the blasting effect is optimal. At this time, the Von Mises stress values corresponding to the three types of mixed emulsion explosives are 31.13 MPa, 28.60 MPa, and 24.37 MPa respectively.

## 5. Conclusions

In this study, the blasting failure process of rock mass under different joint spatial characteristics and explosive parameters is investigated by combining similar model tests with numerical simulations. The effect of joint width, number, spacing, inclination angle, and type of mixed emulsion explosive on the failure degree of rock mass is analyzed in detail. The results of the similar model tests and numerical simulations exhibit excellent consistency, thereby verifying the validity of the result of this study. This study can offer theoretical support and practical guidance for the optimization of dynamic adjustments of blast schemes and safety-risk mitigation in engineering managements. The main conclusions are as follows.

(1) The blasting effect of rock mass first increases and then decreases with the increase of joint width. The average fragmentation size is the smallest when the joint width is 10 mm, and the change in joint width has a relatively small effect on the blasting effect.
(2) The blasting effect of rock mass increases with joint number and spacing. As the joint number and spacing increase, the number and duration of reflected stress waves also increase, causing more fracture surfaces to form in the rock mass, which is conducive to the destruction of the rock mass.
(3) The blasting effect of the rock mass first increases and then decreases with the increase of the joint inclination angle. The average fragmentation size is the smallest when the joint inclination angle is 60°. Under this working condition, more blasting stress waves are obliquely incident and reflected towards the free surface area, and the reflection of the stress waves between the joint surface and the free surface can further enhance the fragmentation effect of the rock mass near the joint surface.
(4) The blasting effect of the H1 mixed emulsion explosive is the best under all working conditions, which means that explosives with high detonation velocity and brisance have greater blasting energy and rock fragmentation ability when the explosive density is the same.
